# Quantitative natural language processing markers of psychoactive drug effects: A pre-registered systematic review

**DOI:** 10.1177/02698811251319455

**Published:** 2025-02-16

**Authors:** Sachin Ahuja, Farida Zaher, Lena Palaniyappan

**Affiliations:** 1 Schulich School of Medicine and Dentistry, University of Western Ontario, London, ON, Canada; 2 Douglas Mental Health University Institute, Montreal, QC, Canada; 3 Department of Psychiatry, McGill University, Montreal, QC, Canada; 4 Robarts Research Institute, University of Western Ontario, London, ON, Canada

**Keywords:** Acoustics, semantics, computational linguistics, intoxication, speech markers

## Abstract

Psychoactive substances used for recreational purposes have mind-altering effects, but systematic evaluation of these effects is largely limited to self-reports. Automated analysis of expressed language (speech and written text) using natural language processing (NLP) tools can provide objective readouts of mental states. In this pre-registered systematic review, we investigate findings from applying the emerging field of computational linguistics to substance use with specific focus on identifying short-term effects of psychoactive drugs. From the literature identified to date, we note that all the studied drugs – stimulants, 3,4-methylenedioxymethamphetamine (MDMA), cannabis, ketamine and psychedelics – affect language production. Based on two or more studies per substance, we note some emerging patterns: stimulants increase verbosity; lysergic acid diethylamide reduces the lexicon; MDMA increases semantic proximity to emotional words; psilocybin increases positive sentiment and cannabis affects speech stream acoustics. Ketamine and other drugs are understudied regarding NLP features (one or no studies). One study provided externally validated support for NLP and machine learning-based identification of MDMA intoxication. We could not undertake a meta-analysis due to the high degree of heterogeneity among outcome measures and the lack of sufficient number of studies. We identify a need for harmonised speech tasks to improve replicability and comparability, standardisation of methods for curating and analysing speech and text data, theory-driven inquiries and the need for developing a shared ‘substance use language corpus’ for data mining. The growing field of computational linguistics can be utilized to advance human behavioral pharmacology of psychoactive substances. Achieving this will require concerted efforts towards consistency in research methods.

## Background

Psychoactive drugs (PADs) including psychedelics/hallucinogens, entactogens, psychostimulants and cannabinoids alter our mental representations and cognition. PADs in these classes are commonly used recreationally and can cause effects that mimic other altered mental states such as psychosis (Brady et al., 1991; Johns, 2001; Krystal et al., 1994). Language, a complex and commonly used cognitive process, is often altered by the acute effects of PADs (Salomé et al., 2000). Nevertheless, linguistic markers are not routinely evaluated when assessing PAD intoxication in clinical, legal or other service settings.

Testing levels of PAD in blood or urine is often used to quantify the amount of consumption (Margolis et al., 2016). For several substances, their systemic levels may not reflect their neurocognitive effects. In other words, a positive test may not translate to observed behaviours (Durback et al., 1998), for various reasons including the individual variations in PAD metabolism (Vizeli et al., 2021). Developing objective markers of the effects of PADs on current mental state would be beneficial for psychiatric assessment in urgent and acute settings, to determine whether a clinical presentation (of, say, psychotic symptoms) is substance-induced.

In research settings, being able to objectively assess the psychoactive effects of a PAD on the mental state would help reduce the overreliance on subjective self-reports, especially as expectancy effects and unblinding are common in this setting (Earleywine et al., 2023; Muthukumaraswamy et al., 2021). Wilful manipulation of one’s speech/writing style to mimic an intoxicated state is possible, but many PADs affect the effortful control of psychomotor functions including language. This makes intentional change of linguistic markers less likely when one is truly under the acute effects of PADs, prompting some to call speech as a ‘window to the intoxicated mind’ (Bedi et al., 2014). Computerised analysis of verbal descriptions obviates the need for introspective self-awareness that is often altered by PADs and provides an alternative with a more empirical quantitative approach that may complement reliability of clinical ratings (Blaabjerg et al., 2020; Espejo et al., 2024; Ross and Leichner, 1988).

By computerised analysis of verbal production, we refer to the automated analysis of expressed language (speech and written text) using natural language processing (NLP) tools. NLP is a subdiscipline of computer science focused on allowing computers to understand and interpret human (i.e. non-programming, ‘natural’) language. NLP methods convert raw natural language data – text or audio of an individual’s language production – into numerical or categorical features and provide various readouts of *how much* is being said (rate and amount of speech, verb density, idea density), *what* is being said (meaning, sentiment and topics conveyed and diversity and similarity among words), and *how* it is being said (the structure of the word sequences, prosody and acoustic aspects of speech) (Le Glaz et al., 2021). From studies that employ human raters, PADs are well established as agents that affect the above aspects of language. For example, ketamine causes disorganised speech resembling psychosis and is termed ‘ketamine-induced thought disorder’ (Adler et al., 1998; Krystal et al., 1994). Furthermore, methamphetamine improves whereas 3,4-methylenedioxymethamphetamine (MDMA) negatively affects verbal fluency, reflecting these PADs’ psychoactive effects on other cognitive processes like attention and memory (Marrone et al., 2010). Finally, cannabis causes speech with greater discontinuity in thought, hypothesised to be caused by cognitive effects such as intruding associations (Roth et al., 1975). A recent review of psychedelics also highlights the value of objective analysis of language production to generate reliable markers of their psychoactive effects (Tagliazucchi, 2022). The quantitative features generated by the NLP analyses can enter machine learning (ML) classifiers to accurately identify if a given speech sample comes from one group (e.g. after cannabis use) or the other (no cannabis use) (Le Glaz et al., 2021).

Here, we review the current literature on how NLP markers are affected by PADs and the accuracy to which classifiers can predict drug status to support the synchronisation of research efforts towards these future applications. Our research questions are:

1. How do PADs including cannabis/cannabinoids, psychostimulants, psychedelics/hallucinogens and entactogens affect language production as measured by NLP markers?
2. How much accuracy has been achieved in predicting a person’s recent use of PADs using computerised language analyses?

To this end, we undertook a pre-registered systematic literature search with a view to meta-analytically combine accuracy metrics provided sufficient number of eligible studies are identified for such statistical pooling. This systematic review followed the Preferred Reporting Items for Systematic Review and Meta-analysis (PRISMA) 2020 guidelines. Details of the protocol for this systematic review were registered on PROSPERO (International prospective register of systematic reviews, Ahuja et al., 2023).

## Methods

### Search strategy and eligibility criteria

For this systematic review, a MEDLINE and Embase search was conducted on 12 June 2023 and 5 March 2024, for articles with terms according to the following two concepts: first, a specific PAD in the psychedelic, hallucinogen, entactogen, stimulant, cannabis or cannabinoid classes, or a related substance-use disorder; and second, a term related to either language or NLP. The specific search terms we utilised are reported in the Supplemental Materials and Methods. Overall, our search found 1051 unique abstracts (discarding duplicates): 961 from the initial search in June 2023, and 90 from the updated search in March 2024. Our eligibility criteria are outlined in Table 1.

**Table 1.** Eligibility criteria.

Inclusion criteria	Exclusion criteria
Participants consumed a drug (or had a diagnosed substance-use disorder) from one of the following PAD categories^a^ at any timepoint before an audio recording or written transcript was collected: psychedelic, hallucinogen, entactogen, stimulant, cannabis or cannabinoid	The study only included patients with a diagnosed psychiatric disorder, or patients with smoking-induced laryngeal changes^b^
There was an audio recording or text transcript of participants’ speech or writing collected in a controlled clinical or research setting	Transcripts were collected from publicly available drug experience repositories, social media or blog posts
The audio recordings or text transcripts were processed using a computer-based analysis	The study only used manual language analyses^c^
	Review studies, conference abstracts and manuscripts in pre-print servers
	The study does not report findings relevant to the effect of PADs on NLP markers^d^

a As classified by a public database on PAD effects (PsychonautWiki Contributors, 2024).

b Lee et al. (1999), Mueller and Wilcox (1980).

c For example manual cloze analyses (Amarel and Cheek, 1965; Honigfeld, 1965; Weil and Zinberg, 1969).

d Alexander-Emery et al. (2005), Cox et al. (2021).

### Study selection and data collection process

**Figure 1.**
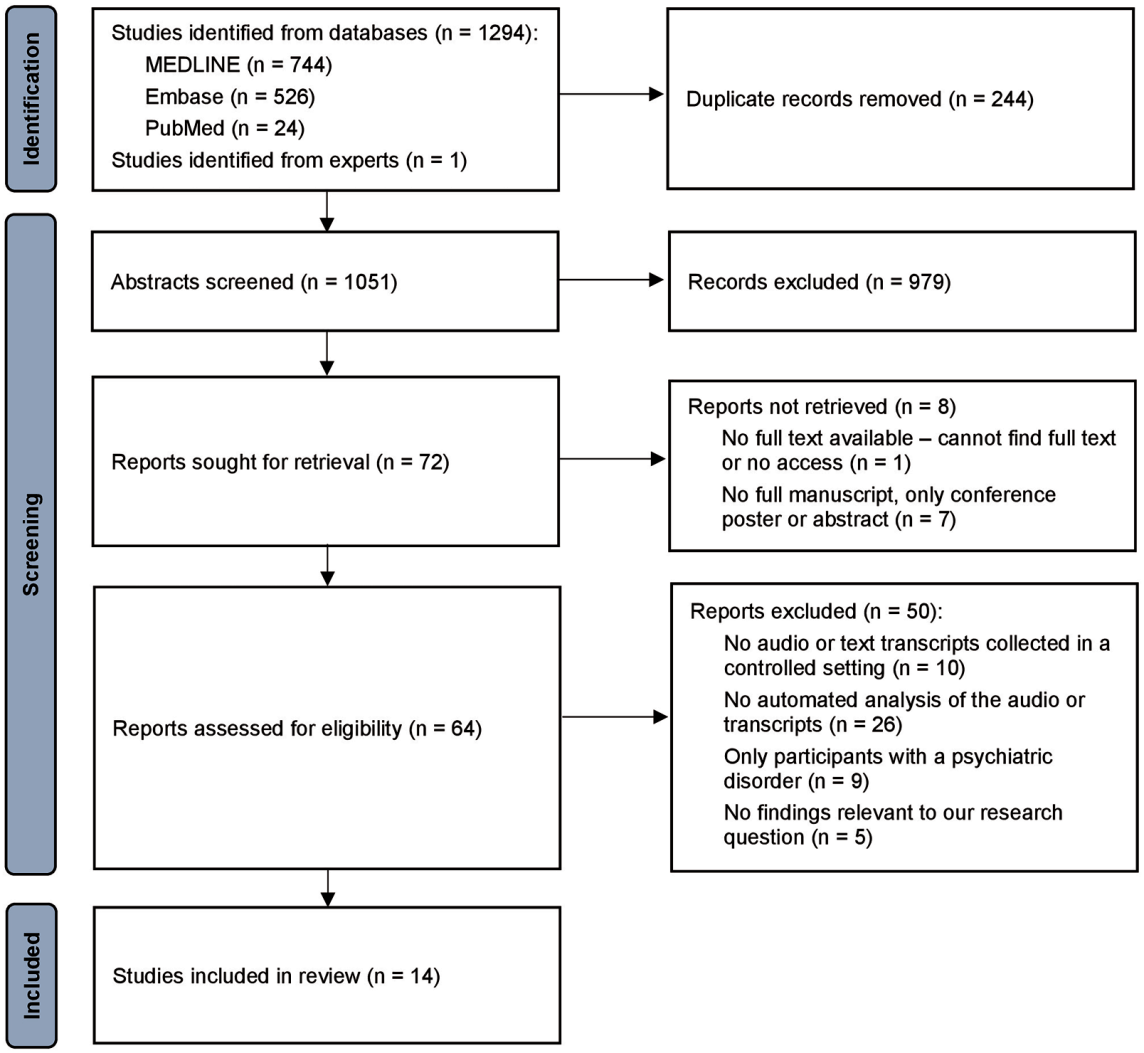
PRISMA flow diagram.

The Covidence software (Covidence, 2023) was used to manage study selection (Figure 1) and data collection. For articles from the first search in June 2023, study screening and subsequent data collection was conducted by two authors independently (SA and FZ), and conflicts were resolved via discussion between the two authors. One author reviewed studies from the updated search in March 2024 (no studies were eligible). The senior author (LP) reviewed the final list independently.

### Data items

The variables extracted from each study included the study aims, recruitment and eligibility criteria, participant demographic variables, study design, drug name and dosing regimen, language task and timing, NLP and classification analyses employed and study results including the effect of study drug on NLP markers and the classification accuracy between study conditions. For variables missing from included publications, the corresponding authors were contacted.

We could not undertake a meta-analysis due to the high degree of heterogeneity among outcome measures and the lack of sufficient number of studies. Therefore, data were synthesised qualitatively for all variables except demographics, which were synthesised quantitatively using the Cochrane Review Manager (Review Manager Web, 2020) and reported as pooled data (Supplemental Table 1). Further details on statistical methods are described in the Supplemental Materials and Methods.

### Risk of bias assessments

Different tools were used to assess risk of bias based on each study’s design: the Risk of Bias in Non-randomized Studies – of Exposures (ROBINS-E) tool was used for cross-sectional studies; the Risk of Bias in Non-randomized Studies – of Interventions (ROBINS-I) tool was used for non-randomised trials; and the Risk of Bias 2 (ROB 2) tool was used for randomised crossover trials. The risk of bias assessments can be found in Supplemental Figure 2.

## Results

**Figure 2.**
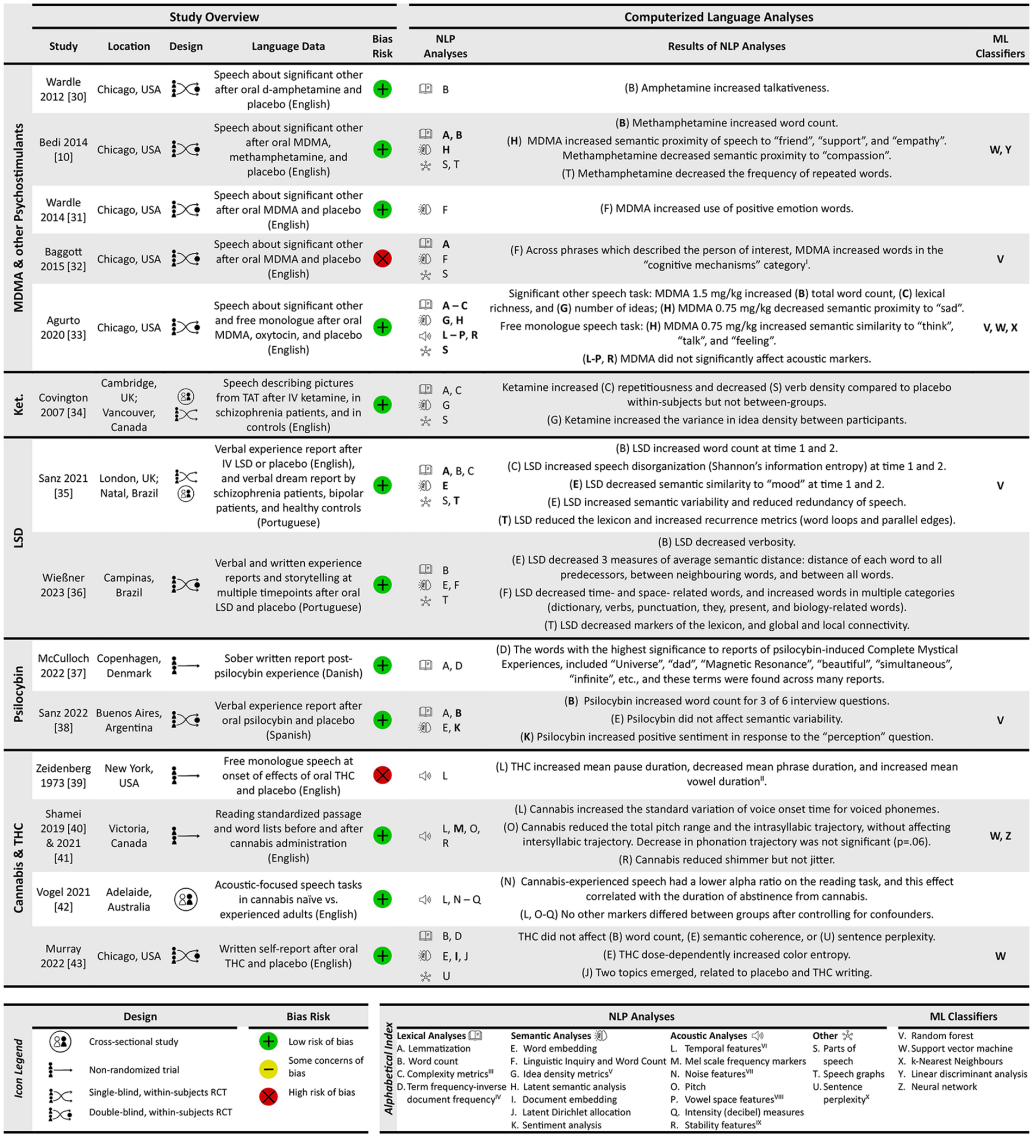
Natural language data source, NLP analyses and findings and ML classifiers used for included studies categorised by PAD. See bottom of figure for icon legend and computer analysis alphabetical index – this categorisation scheme was adapted from Le Glaz et al. (2021). Bold letters are NLP analyses whose results were used as inputs for the study’s classifiers. ^I^After we applied a post hoc Bonferroni adjusted *p*-value cutoff of 0.0012 for 43 hypothesis tests – one for each Linguistic Inquiry and Word Count (LIWC) word category – only this LIWC category was significant. ^II^Significance testing was not performed in the original study, but based on our post hoc paired *t*-tests, all associations were significant (Supplemental Materials and Methods). ^III^Includes metrics related to the total lexicon, such as Honore’s statistic, Brunet and Shannon’s index and Covington and Morris’s perseveration metric. ^IV^Does not include use of LIWC program solely for total word count. ^V^Based on the number of propositions in text estimated using a parts-of-speech (POS) tagging-based method. ^VI^Temporal features such as the distribution of pauses and utterances, or the voice onset time. ^VII^For example, harmonics-to-noise ratio, noise-to-harmonics ratio, mean autocorrelation and alpha ratio of spectral tilt. ^VIII^For example, total area, ‘a-i-u’ area and formants 1,2,3 distribution. ^IX^For example, jitter, shimmer and voice breaks. ^X^Assessed using context-dependent transformer-based approaches (BERT, GPT-2).

Figure 2 provides a summary of the fourteen included studies categorised by PAD. This figure summarises the types of computerised analysis methods used (NLP and ML classifiers), as well as the directional effects of each PAD on NLP markers employed.

### Nature of participants across studies

Across the included studies, there were a total of 406 participants (range 4–71 per study). No participants had a history of psychiatric or substance use disorders, except for comparator subgroups that is, schizophrenia (Covington et al., 2007; Sanz et al., 2021) and bipolar disorder (Sanz et al., 2021) groups. However, participants’ history of prior PAD use varied: previous use of the studied PAD (or related drugs) was required for inclusion in nine studies (Agurto et al., 2020; Baggott et al., 2015; Bedi et al., 2014; Murray and Srinivasa-Desikan, 2022; Shamei and Bird, 2019; Vogel et al., 2021; Wardle et al., 2012; Wardle and De Wit, 2014; Wießner et al., 2023), while individuals with prior psychedelic drug use history were excluded in one study (Sanz et al., 2021).

The mean age of the included sample was 26.8 years (*N* = 375), with 59% of the participants being males. The most represented ethnic group in the sample was White/Caucasian (self-reported) and the language that formed the medium of assessment was mostly English (ten studies) followed by Portuguese (two studies).

### Study designs across drugs

We identified double-blind randomised controlled trial (RCTs; *n* = 8) as the major study type, while single-blind RCTs, non-randomised trials and cross-sectional studies were also reported. While most studies had a within-subject contrast, one study (Vogel et al., 2021) reported only between-subjects data. There were numerous approaches used for eliciting verbal production – two studies relied on written materials (McCulloch et al., 2022; Murray and Srinivasa-Desikan, 2022), while 12 had speech data. When speech was elicited, instructions were highly variable with five studies collecting speech about an important person in the participant’s life (significant other) (Agurto et al., 2020; Baggott et al., 2015; Bedi et al., 2014; Wardle et al., 2012; Wardle and De Wit, 2014), three studies collecting free monologues (Agurto et al., 2020; Vogel et al., 2021; Zeidenberg et al., 1973), three studies collecting experience reports (Sanz et al., 2021, 2022; Wießner et al., 2023), two studies conducting reading tasks (Shamei and Bird, 2019; Vogel et al., 2021) and two studies providing picture prompts (Covington et al., 2007; Wießner et al., 2023). We also noted a wide range of time intervals for language tasks after the cessation of drug intake – 30 min (Covington et al., 2007; Shamei and Bird, 2019) to 24 h (Wießner et al., 2023); while some of this was dictated by the pharmacokinetics of the specific PAD, even within a single class of agent, both the dose (50–75 μg lysergic acid diethylamide [LSD]) and the time interval after administration varied (e.g. 75–180 min for MDMA, 120–225 mins for LSD and 30–100 min for cannabis/THC). Furthermore, the length of time provided for speaking or writing varies between a 5-s sustained vowel production (Vogel et al., 2021) to 10-min speech samples (Agurto et al., 2020; Bedi et al., 2014; Covington et al., 2007; Murray and Srinivasa-Desikan, 2022).

Regarding bias in study designs (Supplemental Figure 2), we noted two studies had higher risk than others based on bias in measurement of outcomes and selection of the reported result (Baggott et al., 2015; Zeidenberg et al., 1973). Consistent with the initial epochs in a field of research, we note an exploratory approach in statistical testing, analysing multiple variables without corrections for inflated type 1 error; for example, 20 or more themes for semantic proximity analysed (in Agurto et al., 2020; Bedi et al., 2014), and 43 variables from Linguistic Inquiry and Word Count (LIWC) analysed (in full transcript and selected parts, Baggott et al., 2015).

### Methods used for speech/text analysis

The most common lexical analyses used were lemmatisation (a pre-processing method used by seven studies) and word count (six studies). A range of semantic analyses were employed that can be broadly grouped under dictionary/corpus-based (LIWC and parts-of-speech (POS) tagging-based idea density), distributional semantics (latent semantic analysis, word embedding and document-embedding models) and topic modelling approaches (Latent Dirichlet allocation and sentiment analysis). Word embedding, the most common semantic analysis (four studies), was done using the Word2Vec (Murray and Srinivasa-Desikan, 2022; Sanz et al., 2021) and FastText (Sanz et al., 2022; Wießner et al., 2023) models. A popular semantic approach used in three studies (Agurto et al., 2020; Bedi et al., 2014; Sanz et al., 2021) was using word embeddings or LSA to test the similarity of the verbal output against a predetermined group of words representing distinct themes of mental states to provide values of *semantic proximity* for each selected theme. Even with this approach, the same set of words were not used for similarity assessment across studies: Bedi et al (2014) used terms for mood states affected by MDMA intoxication, Agurto et al. (2020) added an additional item to this set and Sanz et al. (2021) used terms relevant to LSD-intoxicated speech. Temporal analysis was the most common acoustic approach (three studies). Overall, we found no pair of studies using the same approach for speech or text analysis among our search results.

In the section below, we summarise the drug-specific results reported in more than one study with a view of identifying potential NLP variables for further research. As only one study on ketamine was included (Covington et al., 2007), the effects of ketamine reported in that single study (higher repetitiousness of words with a reduction in verb density) are not discussed any further.

### Replicated drug-specific results

#### Psychostimulants (amphetamine and methamphetamine)

Both Wardle et al. (2012, 20-mg oral *d*-amphetamine, 180-min post-ingestion, 5 mins talking about others) and Bedi et al. (2014, 20-mg oral methamphetamine, 130-min post-ingestion, 10 mins talking about others) reported an increase in talkativeness (word count) with stimulants compared to placebo. This effect was not statistically significant at 10 mg dose of *d*-amphetamine, though no significant differences were reported between the 10 and 20 mg doses. Bedi et al. (2014) also reported a methamphetamine-related reduction in the sequential repetition of the same word (e.g. ‘I. . . I think we are late’), and of the 20 different themes tested for semantic proximity, noted a reduction in words related to ‘compassion’ with methamphetamine compared to placebo. These effects are yet to be replicated. Taken together with studies reporting a significant increase of manually counted syllables 90 mins after oral methamphetamine (Marrone et al., 2010), increased verbosity has been a replicated feature of psychostimulants.

##### 3,4-Methylenedioxymethamphetamine

Bedi et al. (2014) reported an increase in semantic proximity to the positive emotional words ‘friend’ and ‘support’ after 1.5 mg/kg and ‘empathy’ after 0.75 mg/kg of oral MDMA (130-min post-ingestion, 10 mins talking about others), with no changes in verbosity and word repetition patterns in Mota’s graphs, compared to placebo. Agurto et al. (2020) were unable to replicate semantic proximity changes for the same themes despite using a comparable paradigm (0.75 and 1.5 mg/kg oral MDMA, 5 mins talking about others, 75 to 105-min post-ingestion); instead, they reported decreased proximity to ‘sad’ at 0.75 mg/kg dose and increased word count, lexical richness and number of ideas at 1.5 mg/kg dose compared to placebo. Wardle et al. (2014) also used a comparable paradigm in their article (0.75 and 1.5 mg/kg oral MDMA, 5 mins talking about others, 140-min post-ingestion) but did not study semantic proximity. Using dictionary-based word counts, they noted that both doses increased the proportion of positive emotional words, without affecting percentage of negative words used to describe another person. Baggott et al. (2015) used the same task and counting procedure as Wardle et al. but did not see any changes in positive or negative emotion words or overall verbosity 90 mins after MDMA ingestion (1.5 mg/kg). Instead, they noted a decrease in relative terms and motion related words, an increase in words with sexual and social content, words reflecting discrepancies, future and death, and when focusing on phrases that specifically referred to the person being discussed, an increase in references to cognitive processes and insight. Thus, there are no directly replicated results regarding the specific themes affected following MDMA ingestion, though at a broader level, an increase in social and positive emotion content recurs across studies.

##### LSD

Sanz et al. (2021) administered intravenous LSD 75 μg or placebo over two sessions and recorded speech (on the experience of undergoing neuroimaging without time limit) at two time points 120–150 min and 225 min post-infusion. LSD affected the word trajectory structure by increasing verbosity and recurrence along with reduced global connectivity (i.e. long and disconnected with reduced lexicon), affected semantic content by increasing proximity to the word ‘mood’, increasing entropy and semantic variability (reduced coherence). Using a similar experience report approach but with 50 μg oral dose of LSD, Wießner et al. (2023) failed to see any alteration in structure and semantics. But in the same sample, the use of picture description (‘story telling’) revealed *reduced* verbosity and global connectivity with higher recurrence (i.e. short and disconnected with reduced lexicon), affected semantic content with less ‘time’ and ‘space’ related, but more biology-related words, and *decreased* semantic distance between words (more coherence). While there are no directly replicated results on the semantic content and coherence after LSD, reduced lexicon with a disconnected word trajectory has been demonstrated as a post-acute effect in two different tasks, at two different doses.

##### Psilocybin

In Sanz et al.’s (2022) microdosing study, volunteers received placebo or a 0.5 g dose of oral-dried *Psilocybe cubensis* mushrooms (with 0.32 mg psilocybin, 0.48 mg psilocin, 0.025 mg baeocystin and 0.0063 mg norbaeocystin on average) and interviewed 150-min post-ingestion on six topics including feelings, expectation and creativity with no time constraints. Active dose induced higher verbosity especially when asked about mood, alertness and perception, and more positive sentiment when asked about perception, with no changes in semantic variability. While there were no other methodologically similar studies, McCulloch et al. (2022) generated unconstrained, freely written qualitative reports 6–8 h after a medium-high dose (12–30 mg) to compare themes present among those who experienced a Complete Mystical Experience (CME) versus those who did not (groups determined using a 30-item self-report questionnaire). Those who experienced CMEs used words with positive sentiment, relationships and time/space dimensions (e.g. ‘Universe’, ‘dad’, ‘beautiful’, ‘simultaneous’, ‘infinite’, ‘purple’, ‘in relation to’, ‘ray’, ‘happy’ and ‘brother’), while those who did not experience the active dose effect used words with negative sentiment more often (e.g. ‘gloomy’, ‘cycle’, ‘evil’, ‘cold’ and ‘need’). Thus, while there are no directly replicated results on the structure and content of verbal expression after psilocybin use, a preferential expression of positive sentiment has been reported in two studies at least in a subgroup of volunteers.

##### Cannabis and THC

Speech studies on cannabis have generally focussed on acoustic effects but have several methodological shortcomings. The unblinded test–retest designs used by Zeidenberg et al. (1973) included only four participants (male psychiatric resident physicians; 15 mg oral THC, re-tested at 90 mins; 5-min monologue), while Shamei et al. (2021; Shamei and Bird, 2019) included eight (50% females; variable doses, re-tested at 30 mins; reading passages and word lists without time constraint) medicinal cannabis users. These two studies did not test the same speech variables; Zeidenberg et al. (1973) reported increased pause length and vowel duration with reduced phrase duration while Shamei and Bird (2019) reported altered speech stream acoustics (reduced shimmer, pitch range, reduced trajectory of pitch within syllables with increased variability of voice onset time). Vogel et al. (2021) also observed increased pause length with decreased voice onset time in recreational cannabis users without any experimental cannabis administration, indicating a sustained, longer-term effect of cannabis use on speech acoustics.

Murray et al. (2022, oral THC 7.5 mg, 15 mg or placebo administration, 10 mins of written reports 100 mins later) calculated semantic perplexity (unlikelihood of a sentence in a transcript), semantic ‘entropy’ (complement of semantic distance among words in a sentence determined on the basis of 300 dimensional vectors from Word2Vec) and colour ‘entropy’ (semantic distance limited to 8-color dimensions based on 100 images per word, that is, banana is more colour-distant from apple than from lemon). While number of words generated and semantic distance did not change with THC, a dose-dependent increase in ‘colour entropy’ was noted along with a trend level increase in semantic perplexity. Certain words identified using Latent Dirichlet Allocation (LDA)-based topic modelling revealed distinct topics with words ‘time’, ‘moving’, ‘weird’, ‘drug’ and ‘embarrassed’ representing THC-related effects. These methods are yet to be repeated in other samples.

In summary, amidst the methodologically weak studies on the verbal production after cannabis use, there are no directly replicated results; nonetheless, pause length and voice onset times (speech stream markers) appear to be interesting candidates.

### Classification results

In total, 7 of 14 identified studies reported the results of a ML-based classification that aimed to discriminate the speech pattern induced by active drug from placebo. Attempts for external validation has been made only for the MDMA versus placebo contrast, with better-than-chance classification accuracies alongside several limitations in feature selection and sample size, warranting a cautious interpretation.

Bedi et al. (2014) built a Support Vector Machine (SVM) classification model using semantic proximity to ‘rapport’, ‘love’ and ‘support’, as well as word count, and reported an accuracy of 69% in classifying between placebo and methamphetamine-influenced speech, 88% accuracy between MDMA 1.5 mg/kg and placebo and 84% accuracy between MDMA 1.5 mg/kg and methamphetamine (leave one out cross-validations). While speech after MDMA 0.75 mg/kg dose was not clearly separable from placebo, a linear discriminant analysis conducted to classify between all four study conditions and had an accuracy of 59%, with chance corresponding to 25%. Baggott et al. (2015) built a random forest classifier using a bag-of-words approach from the transcripts (using frequency of all words as predictors) and reported an out-of-bag accuracy of 72% for the final model classifying speech after 1.5 mg/kg MDMA from placebo. Agurto et al. (2020) assessed diverse NLP markers (88 acoustic features, 21 semantic proximity concepts (as in Bedi et al., 2014, with the addition of ‘disdain’), and lexical/syntactic features including total word count, number of ideas, propositional density, 6 parts of speech and 2 measures of lexical richness) with SVM and observed accuracies ranging from 71% to 87% (nested leave-one-participant-out cross-validation) when separating speech after MDMA from placebo in the original dataset, with major contributions from semantic proximity (‘anxiety’) and acoustic markers (mel-frequency cepstral coefficients, pitch). When using the same speech NLP features to validate the classifiers on other datasets (Bedi et al., 2014; Wardle and De Wit, 2014), the highest classification accuracies of 66%–92% were found in classifying between MDMA 0.75 mg/kg and placebo.

Sanz et al. (2021) used two random forest classifiers to separate LSD and placebo conditions: one using semantic similarity with ten predefined terms had an area under the curve (AUC) of 0.757, while the one using structural metrics had an AUC of 0.749. For psilocybin versus placebo, Sanz et al. (2022) utilised word count and sentiment scores from six interview questions and built a random forest classifier with stratified cross-validation, reporting an AUC of 0.79. Murray and Srinivasa-Desikan (2022) built an SVM classifier using document embeddings with Term Frequency Inverse Document Frequency (TF-IDF) format and reported an accuracy of 87.5% (AUC of 0.94) in classifying writing under the influence of THC from placebo. Shamei et al. (2021) built SVM, and convolutional neural network (CNN) classifiers using Mel spectrograms from a sustained vowel production task and reported accuracies of 67.2% (female) and 68.2% (male) in classifying cannabis intoxicated speech (gender-specific CNN models with *n*-fold cross-validation; pre-post design).

## Discussion

We reviewed the current literature on how PADs affect NLP markers of verbal expressions and whether intoxication/use status can be accurately predicted with speech and language markers. The result of our systematic search indicates that PADs do affect language production measured by computerised analyses, though consistent patterns required to achieve sufficiently accurate predictions of a person’s recent substance use are not yet available. We discuss the interpretations from the most consistent results, gaps identified in the literature and recommendations for future studies in the sections below.

We found insufficient data and prominent methodological heterogeneity that precluded a pooled synthesis of reported effects across all psychoactive substances reported here. Nonetheless, in more than one study, stimulants (*d*-amphetamine and methamphetamine) increased word count (Bedi et al., 2014; Wardle et al., 2012), in line with the findings from manual analyses of speech (Jaffe et al., 1973; Marrone et al., 2010; Stitzer et al., 1978). Similarly, MDMA seemed to increase social and positive emotion content in speech, consistent with its well-established prosocial effects on subjective mood states (Kamilar-Britt and Bedi, 2015). While these NLP effects do not seem to be dose-dependent (since semantic effects occurred at MDMA 0.75 mg/kg more than 1.5 mg/kg, Agurto et al., 2020), the emerging pattern of MDMA’s effects on sentiment suggests the value of including semantic proximity markers in future studies. Under the influence of LSD (peak effects at 1.5 to 4 h, Dolder et al., 2017), speech becomes more recurrent with a reduction in the use of unique words (Sanz et al., 2021), leading to less connected structure (Wießner et al., 2023). The degree of this disorganisation is limited when compared to the speech of patients with schizophrenia (Mota et al., 2017). Finally, psilocybin appears to increase positive sentiment with ‘microdosing’ (Sanz et al., 2022) and with larger doses in those who report mystical-type psychoactive effects (Garcia-Romeu et al., 2015; Griffiths et al., 2016). Taken together, these observations provide a useful starting point for pursuing automated NLP markers of psychoactive effects that may track their physiological effects in social and clinical settings.

We notice several gaps in the research that aims to apply computational linguistics/NLP in the study of mental state changes induced by PADs. Applying the framework suggested by Miles (2017; Nyanchoka et al., 2019), we tabulate the identified gaps in Table 2.

**Table 2.** Some of the identified gaps in the NLP literature on psychoactive drugs.

Gap domain and contributing observations	Key questions/needs
**Knowledge gap:** Classifier performance of NLP markers not evaluated against human judgement of intoxication; languages other than English not studied, classification not attempted yet for certain drugs	• When assessing intoxication, can objective NLP markers perform better than or provide incremental value to clinical judgement? • Do the NLP markers tracking PAD effects generalise across languages? • Can NLP-based classifiers accurately identify psychostimulant use on an individual basis?
**Evidence gap:** Results from the reviewed studies are contradictory, needing more evidence and multi-method approaches for clarity	• Does verbosity increase in dose-dependent manner with stimulants? Does MDMA affect verbosity in the same way as stimulants? • What is the effect of the duration of interview on semantic and acoustic markers sensitive to PADs? • For each PAD, what speech tasks are the most sensitive to minimum detectable changes?
**Empirical gap:** Replications are lacking for several observations, while other propositions are yet to be evaluated empirically	• Does ketamine affect repetitiousness and idea density as reported? • Do stimulants reduce sequential word repeats and reduce ‘compassion’ in speech? • Does LSD reduce verbosity? Is ‘storytelling’ superior to ‘experience reporting’ to elicit LSD-related speech changes? • Does cannabis affect semantic coherence/perplexity? Is cannabis-induced alteration in speech stream a long-term effect that persists beyond its peak physiological effects? • Does microdosing with psilocybin affect speech output for selected themes only?
**Population gap:** Clinical populations for whom end results are applicable are under-researched	• Do NLP markers change in persons with SUD to the same extent as in healthy volunteers with experimental exposure? • Are the effects of PADs sufficiently distinct from the effect of disorders such as schizophrenia, depression and mania? • Some speech markers are more sensitive than others to the social background (race/ethnicity, cultural background, socioeconomic status). How does this affect the drug effect?
**Theoretical gap:** Pharmacological and cognitive theories not being applied in variable selection	• Cannabis may induce paranoia by increasing self-relevance of stimuli. Does semantic content reflect this effect? • As pharmacological response shows inter-individual variations, can we identify clusters/subgroups of subjects with varying speech patterns using unsupervised classifiers?
**Methodological gap:** Lack of control arm and blinding is common, dialogical/social interactions with two or more people remain unstudied	• Are speech changes induced by cannabis observable in double-blind RCT design? • What is the influence of interviewer’s facilitations in sentiment and content analysis reported in MDMA, LSD and psilocybin studies?
**Action gap:** Observed research practices deviate from perspectives gained from other fields	• Need to attempt replication of prior studies when new experiments are designed • Need for overlapping protocols of language data acquisition affecting comparisons across substances • Need for accessible/shared corpora to enable external validation of classifiers • Lack of structured reporting of data capture, curation, processing pipelines, model selection, parameter tuning and algorithmic decisions • Missed opportunities of collecting language samples during therapeutic trials (e.g. MDMA in PTSD, psychedelics/ketamine in depression)

SUD: substance use disorders; RCT: randomised controlled trial; PTSD: post-traumatic stress disorder; PAD: psychoactive drug; NLP: natural language processing; MDMA: 3,4-methylenedioxymethamphetamine.

Several directions for future studies emerge from this work. Some of these are tabulated in Table 2. Classification accuracies of drug status are often above chance and have been validated using external cohorts in the case of Agurto et al. (2020). Nevertheless, classifiers have large variation in accuracy across studies, with no replication studies of model accuracy. There is an urgent need for the use of a shared protocol for speech data collection, deriving consensus on experimental parameters (e.g. post-ingestion timing of acquisition, recording equipment standards, pre-processing routines) and collaborative sharing of recorded speech samples to enable systematic studies of the effects of various NLP analytical pipelines. Tagliazucchi (2022) recommended that researchers conducting therapeutic trials of PADs routinely collect natural language samples and analyse them using NLP to validate the psychometric properties of the NLP markers. Additionally, we recommend that ongoing studies where speech is being collected as a by-product of planned assessments analyse this speech data using NLP. Future studies should attempt to replicate promising NLP findings such as those identified in this review to accelerate clinical translation. Future work on PAD-induced speech effects should also explore other PADs, study designs, dosing regimens, languages, speech tasks and NLP markers (such as markers known to be affected in psychosis (Corcoran et al., 2020) and depression (Koops et al., 2021)) while keeping the most likely use cases for a speech-based ML classifier in mind. For speech recordings to be used in the identification of an individual’s substance use status in clinical settings, designing studies that compare one PAD against another, as well as against other altered mental states such as psychosis or mood episodes, are essential.

A limitation of the current pre-registered review is that it does not include several psychoactive substances that may affect speech, but are less likely to present with a particular challenge in identifying states of intoxication, and have limited NLP research to date (e.g. depressants, neuroleptics). Also, a numerical synthesis of effect size differences or classification accuracies was not attempted due the high degree of methodological heterogeneity. Given these issues, we urge caution when interpreting the findings on drug-specific NLP changes reported in our review. Our observations are best considered as a starting point for further experimental work in this field.

In summary, language production appears to be consistently affected by PADs. Objective, computerised analyses appear sensitive to detect these changes. With continued efforts towards investigating promising NLP markers and open questions in the field, speech - a highly accessible and non-invasive (Palaniyappan et al., 2022) source of biosocial markers (Palaniyappan, 2021) - can be leveraged to expand human behavioural pharmacology.

## Supplemental Material

sj-docx-1-jop-10.1177_02698811251319455 – Supplemental material for Quantitative natural language processing markers of psychoactive drug effects: A pre-registered systematic review Supplemental material, sj-docx-1-jop-10.1177_02698811251319455 for Quantitative natural language processing markers of psychoactive drug effects: A pre-registered systematic review by Sachin Ahuja, Farida Zaher and Lena Palaniyappan in Journal of Psychopharmacology

sj-docx-2-jop-10.1177_02698811251319455 – Supplemental material for Quantitative natural language processing markers of psychoactive drug effects: A pre-registered systematic review Supplemental material, sj-docx-2-jop-10.1177_02698811251319455 for Quantitative natural language processing markers of psychoactive drug effects: A pre-registered systematic review by Sachin Ahuja, Farida Zaher and Lena Palaniyappan in Journal of Psychopharmacology

sj-docx-3-jop-10.1177_02698811251319455 – Supplemental material for Quantitative natural language processing markers of psychoactive drug effects: A pre-registered systematic review Supplemental material, sj-docx-3-jop-10.1177_02698811251319455 for Quantitative natural language processing markers of psychoactive drug effects: A pre-registered systematic review by Sachin Ahuja, Farida Zaher and Lena Palaniyappan in Journal of Psychopharmacology

sj-docx-4-jop-10.1177_02698811251319455 – Supplemental material for Quantitative natural language processing markers of psychoactive drug effects: A pre-registered systematic review Supplemental material, sj-docx-4-jop-10.1177_02698811251319455 for Quantitative natural language processing markers of psychoactive drug effects: A pre-registered systematic review by Sachin Ahuja, Farida Zaher and Lena Palaniyappan in Journal of Psychopharmacology
