# Supplementary material for: Quantitative natural language processing markers of psychoactive drug effects: A pre-registered systematic review
Source: J Psychopharmacol. 2025 Feb 16;39(9):940–9. doi: 10.1177/02698811251319455 (PMC12371134; doi:10.1177/02698811251319455)
Supplement: sj-docx-1-jop-10.1177_02698811251319455 – Supplemental material for Quantitative natural language processing markers of psychoactive drug effects: A pre-registered systematic review [file sj-docx-1-jop-10.1177_02698811251319455.docx]

**Supplementary Table 1** Pooled Demographic Characteristics

|  | **N of studies^I^** | **N of participants** | **Mean (95% CI)** |
| --- | --- | --- | --- |
| **Age** | 11 | 375 | 26.8 (25.0 – 28.6) |
| **% female** | 13 | 387 | 160 (41%) |
| **Race** | 6 | 176 |  |
| White^II^ |  |  | 140 (80%) |
| Black^III^ |  |  | 12 (7%) |
| Asian |  |  | 4 (2%) |
| Other^IV^ |  |  | 20 (11%) |
| **Years of Education** | 7 | 225 | 16.0 (15.2 – 16.8) |

*A random-effect model was used to pool participants’ mean ages and years of education. For cohorts that were used in multiple publications (e.g. as the training cohort in one study and the validation cohort in another study* [3,4]*), those participants were only counted once. ^I^Number of studies with data fit for pooled analysis;* ^II^*or “Caucasian”;* ^III^*or “African American”;* ^IV^*other and/or mixed race.*
