# Supplementary material for: Quantitative natural language processing markers of psychoactive drug effects: A pre-registered systematic review
Source: J Psychopharmacol. 2025 Feb 16;39(9):940–9. doi: 10.1177/02698811251319455 (PMC12371134; doi:10.1177/02698811251319455)
Supplement: sj-docx-2-jop-10.1177_02698811251319455 – Supplemental material for Quantitative natural language processing markers of psychoactive drug effects: A pre-registered systematic review [file sj-docx-2-jop-10.1177_02698811251319455.docx]

Supplementary Materials and Methods

Table of Contents

Page 1: Supplementary Analyses

Page 2: Risk of bias assessments

Page 3-8: Search Strategy

Page 9: References

supplementary Analyses

The pooled demographic characteristics of participants in the included studies are presented in Supplementary Table 1.

**Supplementary Table 1** Pooled Demographic Characteristics

|  | **N of studies^I^** | **N of participants** | **Mean (95% CI)** |
| --- | --- | --- | --- |
| **Age** | 11 | 375 | 26.8 (25.0 – 28.6) |
| **% female** | 13 | 387 | 160 (41%) |
| **Race** | 6 | 176 |  |
| White^II^ |  |  | 140 (80%) |
| Black^III^ |  |  | 12 (7%) |
| Asian |  |  | 4 (2%) |
| Other^IV^ |  |  | 20 (11%) |
| **Years of Education** | 7 | 225 | 16.0 (15.2 – 16.8) |

*A random-effect model was used to pool participants’ mean ages and years of education. For cohorts that were used in multiple publications (e.g. as the training cohort in one study and the validation cohort in another study (Bedi et al., 2014; Wardle and De Wit, 2014), those participants were only counted once. ^I^Number of studies with data fit for pooled analysis;* ^II^*or “Caucasian”;* ^III^*or “African American”;* ^IV^*other and/or mixed race.*

In the study by Zeidenberg and colleagues (Zeidenberg et al., 1973), no significance testing was performed, thus we performed post-hoc paired t-tests (one-tailed for pause duration and phrase duration according to Zeidenberg and colleagues’ hypotheses and two-tailed for vowel duration) between placebo speech (time 1) and THC-influenced speech (time 2) using GraphPad Prism (GraphPad Prism, 2024), which showed significant changes for all associations (Figure 2). We found a significant increase in mean pause duration from placebo (mean 0.793 sec (SD 0.496)) to THC-influenced speech (mean 1.074 sec (SD 0.262)) (p = 0.030); a significant decrease in mean phrase duration from placebo (mean 1.490 sec (SD 0.262)) to THC-influenced speech (mean 1.238 sec (SD 0.151)) (p = 0.027); and a significant increase in mean vowel duration from placebo (mean 0.197 sec (SD 0.041)) to THC-influenced speech (mean 0.221 sec (SD 0.049)) (p = 0.048).

Risk of Bias Assessments

**Supplementary Figure 1** Risk of bias assessments


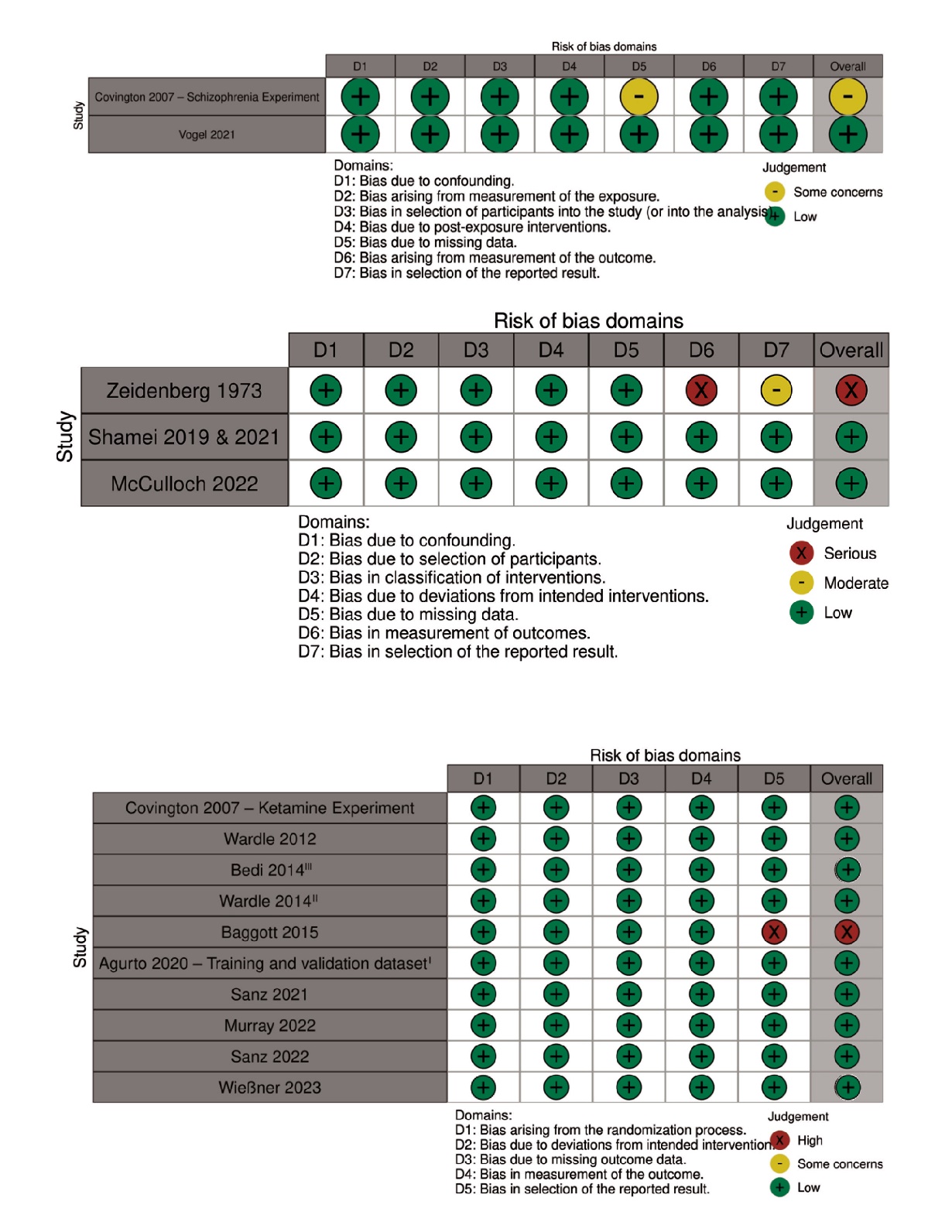


*From top to bottom: risk of bias for cross-sectional studies, risk of bias for non-randomized trials, and risk of bias for randomized crossover trials included in the review. ^I^For Agurto et al., 2020, we report the risk of bias for the main cohort of 31 healthy adults; ^II^Wardle and De Wit, 2014 and ^III^Bedi et al., 2014 are both independently included in the review, but the data from these studies were also used in Agurto et al., 2020 as “Independent Datasets 1 and 2” for model testing.*

Search Strategy

The specific terms that were used to search MEDLINE and Embase databases are reported below. The search terms were created in consultation with a professional healthcare librarian, and a study by Sanz et al. (2018) was utilized as a reference of 165 potentially relevant psychoactive drugs to be included in the study.

## **Ovid MEDLINE search terms**

1: exp Hallucinogens/; 2: Lysergic Acid Diethylamide/; 3: Psilocybin/; 4: exp N,N-Dimethyltryptamine/; 5: Mescaline/; 6: Ketamine/; 7: N-Methyl-3,4-methylenedioxyamphetamine/; 8: 3,4-methylenedioxyamphetamine/ or n-methyl-3,4-methylenedioxyamphetamine/; 9: Ibogaine/; 10: Phencyclidine/; 11: Harmine/; 12: Dextromethorphan/; 13: Central Nervous System Stimulants/; 14: cocaine/ or crack cocaine/; 15: Cocaine Smoking/; 16: Cocaine-Related Disorders/; 17: Amphetamines/; 18: Amphetamine-Related Disorders/; 19: Amphetamine/; 20: Dextroamphetamine/; 21: Lisdexamfetamine Dimesylate/; 22: Methamphetamine/; 23: Benzphetamine/; 24: p-Chloroamphetamine/; 25: phentermine/ or chlorphentermine/; 26: Nicotine/; 27: "Tobacco Use Disorder"/; 28: Caffeine/; 29: Methylphenidate/; 30: Dexmethylphenidate Hydrochloride/; 31: Ephedrine/; 32: Harmaline/; 33: Mazindol/; 34: Pemoline/; 35: Phenmetrazine/; 36: synthetic cathinone/; 37: Myristica/; 38: Cannabis/; 39: Marijuana Abuse/; 40: exp Cannabinoids/; 41: Hallucinogen*.tw,kf.; 42: Psychedel*.tw,kf.; 43: Psychodel*.tw,kf.; 44: lysergic acid diethylamide.tw,kf.; 45: delysid.tw,kf.; 46: lysergic acid d diethylamide.tw,kf.; 47: n,n diethyllysergamide.tw,kf.; 48: psilocybin.tw,kf.; 49: psilocin.tw,kf.; 50: psilocybine.tw,kf.; 51: indocybin.tw,kf.; 52: n,n-dimethyltryptamine.tw,kf.; 53: n dimethyltryptamine.tw,kf.; 54: dimethyltryptamine.tw,kf.; 55: dimethyl tryptamine.tw,kf.; 56: n,n dimethyl tryptamine.tw,kf.; 57: methoxydimethyltryptamine*.tw,kf.; 58: Ayahuasca.tw,kf.; 59: Psychotria viridis.tw,kf.; 60: Anadenanthera peregrina.tw,kf.; 61: mescalin*.tw,kf.; 62: peyote.tw,kf.; 63: peyotl.tw,kf.; 64: Lophophora williamsii.tw,kf.; 65: trimethoxyphenethylamine.tw,kf.; 66: Ketamine.tw,kf.; 67: Esketamine.tw,kf.; 68: spravato.tw,kf.; 69: katamine.tw,kf.; 70: ketolar.tw,kf.; 71: S-ketamine.tw,kf.; 72: R-ketamine.tw,kf.; 73: Arketamine.tw,kf.; 74: MDMA.tw,kf.; 75: n methyl 3,4 methylenedioxyamphetamine.tw,kf.; 76: ecstasy.tw,kf.; 77: methylenedioxymethamphetamine.tw,kf.; 78: 3,4-Methylenedioxymethamphetamine.tw,kf.; 79: MDMA-assisted psychotherapy.tw,kf.; 80: 3,4 methylenedioxyamphetamine.tw,kf.; 81: methylene dioxyamphetamine.tw,kf.; 82: methylenedioxyamphetamine.tw,kf.; 83: Ibogaine.tw,kf.; 84: Iboga.tw,kf.; 85: endabuse.tw,kf.; 86: 5-MeO-DMT.tw,kf.; 87: Methoxydimethyltryptamine*.tw,kf.; 88: Entactogen*.tw,kf.; 89: 5 methoxy n,n dimethyltryptamine.tw,kf.; 90: 5 methoxydimethyltryptamine.tw,kf.; 91: 5 methoxy dmt.tw,kf.; 92: Salvia divinorum.tw,kf.; 93: Diviners sage.tw,kf.; 94: Phencyclidine.tw,kf.; 95: Phencyclidin.tw,kf.; 96: phenycyclidine.tw,kf.; 97: phenylcyclidine.tw,kf.; 98: Angel dust.tw,kf.; 99: sernyl.tw,kf.; 100: Bufotenin.tw,kf.; 101: Bufotenine.tw,kf.; 102: dimethylserotonin.tw,kf.; 103: 2C-B.tw,kf.; 104: 2C-I.tw,kf.; 105: 2C-E.tw,kf.; 106: 2C-T-7.tw,kf.; 107: 2C-T-2.tw,kf.; 108: 2C-C.tw,kf.; 109: 2C-P.tw,kf.; 110: 2C-T-4.tw,kf.; 111: Argyreia nervosa.tw,kf.; 112: Diisopropyltryptamine.tw,kf.; 113: 5 methoxy n,n diisopropyltryptamine.tw,kf.; 114: foxy methoxy.tw,kf.; 115: 4 iodo 2,5 dimethoxyphenylisopropylamine.tw,kf.; 116: Methylone.tw,kf.; 117: 2,5-Dimethoxy-4-Methylamphetamine.tw,kf.; 118: 25I-NBOMe.tw,kf.; 119: 5-MeO-aMT.tw,kf.; 120: 4-AcO-DMT.tw,kf.; 121: 5-MeO-MiPT.tw,kf.; 122: Bromo-DragonFLY.tw,kf.; 123: AL-LAD.tw,kf.; 124: Central Nervous System Stimulant*.tw,kf.; 125: cns stimulant*.tw,kf.; 126: Central stimulant*.tw,kf.; 127: Stimulant-use disorder*.tw,kf.; 128: Stimulant-related disorder*.tw,kf.; 129: Psychostimulant.tw,kf.; 130: Cocaine.tw,kf.; 131: Crack cocaine.tw,kf.; 132: cocain.tw,kf.; 133: Cocaine abuse.tw,kf.; 134: Cocaine addiction.tw,kf.; 135: Cocaine dependen*.tw,kf.; 136: Cocaine related disorder*.tw,kf.; 137: Cocaine-use disorder.tw,kf.; 138: Amphetamines.tw,kf.; 139: Amphetamine-related disorder*.tw,kf.; 140: Amphetamine abuse.tw,kf.; 141: Amphetamine addiction.tw,kf.; 142: Amphetamine dependen*.tw,kf.; 143: Amphetamine-use disorder*.tw,kf.; 144: Amphetamine.tw,kf.; 145: Amfetamine.tw,kf.; 146: Adderall.tw,kf.; 147: levoamphetamine.tw,kf.; 148: adzenys.tw,kf.; 149: Amphetamin.tw,kf.; 150: amphethamine.tw,kf.; 151: Benzedrine.tw,kf.; 152: centramine.tw,kf.; 153: evekeo.tw,kf.; 154: levamfetamine.tw,kf.; 155: levamphetamine.tw,kf.; 156: levoamphetamine.tw,kf.; 157: Methamphetamine.tw,kf.; 158: crystal meth.tw,kf.; 159: deoxyephedrine.tw,kf.; 160: Desoxyephedrine.tw,kf.; 161: desoxyn.tw,kf.; 162: metamfetamine.tw,kf.; 163: methylamphetamine.tw,kf.; 164: methedrine.tw,kf.; 165: pervitin.tw,kf.; 166: Methamphetamine dependenc*.tw,kf.; 167: methamphetamine addiction.tw,kf.; 168: Dextroamphetamine.tw,kf.; 169: Dexamfetamine.tw,kf.; 170: Dexedrine.tw,kf.; 171: dextro amphetamine sulfate.tw,kf.; 172: dextrostat.tw,kf.; 173: Lisdexamfetamine Dimesylate.tw,kf.; 174: elvanse.tw,kf.; 175: lisdexamfetamine.tw,kf.; 176: Vyvanse.tw,kf.; 177: elvanse.tw,kf.; 178: venvanse.tw,kf.; 179: parachloroamphetamine.tw,kf.; 180: chloramphetamine.tw,kf.; 181: chloroamphetamine.tw,kf.; 182: 4-Fluoroamphetamine.tw,kf.; 183: Nicotine.tw,kf.; 184: Nicotine bitartrate.tw,kf.; 185: nicotine tartrate.tw,kf.; 186: Tobacco.tw,kf.; 187: habitrol.tw,kf.; 188: nicabate.tw,kf.; 189: nicoderm.tw,kf.; 190: nicopass.tw,kf.; 191: nicopatch.tw,kf.; 192: Nicorette.tw,kf.; 193: nicotin.tw,kf.; 194: nicotinell.tw,kf.; 195: nicotrol.tw,kf.; 196: niquitin.tw,kf.; 197: Nicotine-use disorder.tw,kf.; 198: Tobacco-use disorder.tw,kf.; 199: Nicotine addiction*.tw,kf.; 200: Tobacco addiction*.tw,kf.; 201: Nicotine dependen*.tw,kf.; 202: Tobacco dependen*.tw,kf.; 203: Nicotine abuse.tw,kf.; 204: Tobacco abuse.tw,kf.; 205: Caffeine.tw,kf.; 206: Coffee.tw,kf.; 207: Yerba mate.tw,kf.; 208: Ilex paraguariensis.tw,kf.; 209: vivarin.tw,kf.; 210: caffein.tw,kf.; 211: percoffedrinol.tw,kf.; 212: coffeine.tw,kf.; 213: Methylphenidate.tw,kf.; 214: centedrin.tw,kf.; 215: concerta.tw,kf.; 216: daytrana.tw,kf.; 217: equasym.tw,kf.; 218: metadate.tw,kf.; 219: methylin.tw,kf.; 220: phenidylate.tw,kf.; 221: Ritalin.tw,kf.; 222: ritalina.tw,kf.; 223: ritaline.tw,kf.; 224: rubifen.tw,kf.; 225: adhansia.tw,kf.; 226: aptensio.tw,kf.; 227: biphentin.tw,kf.; 228: cotempla.tw,kf.; 229: foquest.tw,kf.; 230: jornay.tw,kf.; 231: medicebran.tw,kf.; 232: medikinet.tw,kf.; 233: methylfenidate.tw,kf.; 234: methylphenidylacetate.tw,kf.; 235: methylphenydate.tw,kf.; 236: "prc 063".tw,kf.; 237: quasym.tw,kf.; 238: quillichew.tw,kf.; 239: quillivant.tw,kf.; 240: Dexmethylphenidate.tw,kf.; 241: dexmethylphenidate hydrochloride.tw,kf.; 242: focalin.tw,kf.; 243: ritadex.tw,kf.; 244: mazanor.tw,kf.; 245: mazindol.tw,kf.; 246: sanorex.tw,kf.; 247: phenoxazole.tw,kf.; 248: tradon.tw,kf.; 249: phenmetrazine.tw,kf.; 250: preludin.tw,kf.; 251: dexphenmetrazine.tw,kf.; 252: Cathinone.tw,kf.; 253: Khat.tw,kf.; 254: Synthetic Cathinone*.tw,kf.; 255: Psychoactive bath salts.tw,kf.; 256: Methylenedioxypyrovalerone.tw,kf.; 257: MDPV.tw,kf.; 258: Katinon.tw,kf.; 259: Catinon.tw,kf.; 260: Propylhexedrine.tw,kf.; 261: Benzedrex.tw,kf.; 262: Dristan Inhaler.tw,kf.; 263: Obesin.tw,kf.; 264: Mephedrone.tw,kf.; 265: Ethylphenidate.tw,kf.; 266: cannabi*.tw,kf.; 267: cannabis.tw,kf.; 268: bhang.tw,kf.; 269: hashish*.tw,kf.; 270: marihuana*.tw,kf.; 271: marijuana*.tw,kf.; 272: medical marihuana*.tw,kf.; 273: Cannabis-use disorder.tw,kf.; 274: Marijuana-use disorder.tw,kf.; 275: Cannabis abuse*.tw,kf.; 276: Marijuana abuse*.tw,kf.; 277: Cannabis dependenc*.tw,kf.; 278: Marijuana dependenc*.tw,kf.; 279: Cannabis addiction.tw,kf.; 280: Marijuana addiction.tw,kf.; 281: Anandamide.tw,kf.; 282: Cannabinol.tw,kf.; 283: Delta8-tetrahydrocannabinol.tw,kf.; 284: Delta9-tetrahydrocannabinol.tw,kf.; 285: Dronabinol.tw,kf.; 286: Levonantradol.tw,kf.; 287: Methanandamide.tw,kf.; 288: Nabiximol*.tw,kf.; 289: Nantradol.tw,kf.; 290: Tetrahydrocannabinol.tw,kf.; 291: Tetrahydrocannabinol derivative*.tw,kf.; 292: THC.tw,kf.; 293: Delta-9-THC.tw,kf.; 294: Delta-9-tetrahydrocannabinol.tw,kf.; 295: Delta-1-THC.tw,kf.; 296: Delta-1-tetrahydrocannabinol.tw,kf.; 297: 1 or 2 or 3 or 4 or 5 or 6 or 7 or 8 or 9 or 10 or 11 or 12 or 13 or 14 or 15 or 16 or 17 or 18 or 19 or 20 or 21 or 22 or 23 or 24 or 25 or 26 or 27 or 28 or 29 or 30 or 31 or 32 or 33 or 34 or 35 or 36 or 37 or 38 or 39 or 40 or 41 or 42 or 43 or 44 or 45 or 46 or 47 or 48 or 49 or 50 or 51 or 52 or 53 or 54 or 55 or 56 or 57 or 58 or 59 or 60 or 61 or 62 or 63 or 64 or 65 or 66 or 67 or 68 or 69 or 70 or 71 or 72 or 73 or 74 or 75 or 76 or 77 or 78 or 79 or 80 or 81 or 82 or 83 or 84 or 85 or 86 or 87 or 88 or 89 or 90 or 91 or 92 or 93 or 94 or 95 or 96 or 97 or 98 or 99 or 100 or 101 or 102 or 103 or 104 or 105 or 106 or 107 or 108 or 109 or 110 or 111 or 112 or 113 or 114 or 115 or 116 or 117 or 118 or 119 or 120 or 121 or 122 or 123 or 124 or 125 or 126 or 127 or 128 or 129 or 130 or 131 or 132 or 133 or 134 or 135 or 136 or 137 or 138 or 139 or 140 or 141 or 142 or 143 or 144 or 145 or 146 or 147 or 148 or 149 or 150 or 151 or 152 or 153 or 154 or 155 or 156 or 157 or 158 or 159 or 160 or 161 or 162 or 163 or 164 or 165 or 166 or 167 or 168 or 169 or 170 or 171 or 172 or 173 or 174 or 175 or 176 or 177 or 178 or 179 or 180 or 181 or 182 or 183 or 184 or 185 or 186 or 187 or 188 or 189 or 190 or 191 or 192 or 193 or 194 or 195 or 196 or 197 or 198 or 199 or 200 or 201 or 202 or 203 or 204 or 205 or 206 or 207 or 208 or 209 or 210 or 211 or 212 or 213 or 214 or 215 or 216 or 217 or 218 or 219 or 220 or 221 or 222 or 223 or 224 or 225 or 226 or 227 or 228 or 229 or 230 or 231 or 232 or 233 or 234 or 235 or 236 or 237 or 238 or 239 or 240 or 241 or 242 or 243 or 244 or 245 or 246 or 247 or 248 or 249 or 250 or 251 or 252 or 253 or 254 or 255 or 256 or 257 or 258 or 259 or 260 or 261 or 262 or 263 or 264 or 265 or 266 or 267 or 268 or 269 or 270 or 271 or 272 or 273 or 274 or 275 or 276 or 277 or 278 or 279 or 280 or 281 or 282 or 283 or 284 or 285 or 286 or 287 or 288 or 289 or 290 or 291 or 292 or 293 or 294 or 295 or 296; 298: Natural Language Processing/; 299: Sentiment Analysis/; 300: Natural Language Process*.tw,kf.; 301: Language Process*.tw,kf.; 302: Sentiment Analys*.tw,kf.; 303: Semantic Analys*.tw,kf.; 304: Linguistic Analys*.tw,kf.; 305: Keyword extract*.tw,kf.; 306: Part-of-speech.tw,kf.; 307: Parts-of-speech.tw,kf.; 308: Parts of speech.tw,kf.; 309: POS tag*.tw,kf.; 310: 298 or 299 or 300 or 301 or 302 or 303 or 304 or 305 or 306 or 307 or 308 or 309; 311: Speech/; 312: speech acoustics/; 313: Speech Recognition Software/; 314: Narration/; 315: exp Voice/; 316: Verbal Behavior/; 317: linguistics/; 318: phonetics/; 319: psycholinguistics/; 320: semantics/; 321: vocabulary/; 322: Writing/; 323: sound recordings/; 324: 310 or 311 or 312 or 313 or 314 or 315 or 316 or 317 or 318 or 319 or 320 or 321 or 322 or 323; 325: 297 and 324;

## **Ovid Embase search terms**

1: exp psychedelic agent/; 2: hallucinogenic plant/; 3: hallucinogenic fungus/; 4: lysergide/; 5: psilocybine/; 6: n,n dimethyltryptamine/; 7: mescaline/; 8: ketamine/; 9: midomafetamine/; 10: 3,4 methylenedioxyamphetamine/; 11: ibogaine/; 12: salvia divinorum/; 13: 5 methoxy n,n dimethyltryptamine/; 14: phencyclidine/; 15: bufotenine/; 16: harmine/; 17: dextromethorphan/; 18: ipomoea/ or ipomoea purpurea/; 19: n,n diisopropyl 5 methoxytryptamine/; 20: brolamfetamine/; 21: 4 iodo 2,5 dimethoxyamphetamine/; 22: methylone/; 23: n ethyl 3,4 methylenedioxyamphetamine/; 24: central stimulant agent/; 25: cocaine/; 26: cocaine dependence/; 27: amphetamine derivative/; 28: amphetamine dependence/; 29: amphetamine/; 30: dexamphetamine/; 31: lisdexamfetamine/; 32: methamphetamine/; 33: methamphetamine dependence/; 34: benzphetamine/; 35: chloramphetamine/; 36: phentermine/; 37: chlorphentermine/; 38: nicotine/; 39: tobacco dependence/; 40: caffeine/; 41: methylphenidate/; 42: dexmethylphenidate/; 43: ephedrine/; 44: harmaline/; 45: mazindol/; 46: pemoline/; 47: phenmetrazine/; 48: cathinone/; 49: nutmeg/; 50: adrafinil/; 51: exp psychostimulant agent/; 52: propylhexedrine/; 53: 4' methylmethcathinone/; 54: cannabis/; 55: cannabis sativa/; 56: medical cannabis/; 57: cannabis derivative/; 58: "cannabis (genus)"/; 59: "cannabis sativa subsp. indica"/; 60: "cannabis sativa subsp. sativa"/; 61: cannabis addiction/; 62: cannabinoid/ or anandamide/ or cannabichromene/ or cannabidiol/ or cannabidiol derivative/ or delta8 tetrahydrocannabinol/ or delta8 tetrahydrocannabinol derivative/ or "delta9(11) tetrahydrocannabinol"/ or dexanabinol/ or dronabinol/ or endocannabinoid/ or levonantradol/ or methanandamide/ or n oleoylethanolamine/ or nabiximols/ or nantradol/ or noladin ether/ or tetrahydrocannabinol/ or tetrahydrocannabinol derivative/ or tetrahydrocannabinolic acid/ or virodhamine/; 63: Hallucinogen*.tw,kf.; 64: Psychedel*.tw,kf.; 65: Psychodel*.tw,kf.; 66: lysergic acid diethylamide.tw,kf.; 67: delysid.tw,kf.; 68: lysergic acid d diethylamide.tw,kf.; 69: n,n diethyllysergamide.tw,kf.; 70: psilocybin.tw,kf.; 71: psilocin.tw,kf.; 72: psilocybine.tw,kf.; 73: indocybin.tw,kf.; 74: n,n-dimethyltryptamine.tw,kf.; 75: n dimethyltryptamine.tw,kf.; 76: dimethyltryptamine.tw,kf.; 77: dimethyl tryptamine.tw,kf.; 78: n,n dimethyl tryptamine.tw,kf.; 79: methoxydimethyltryptamine*.tw,kf.; 80: Ayahuasca.tw,kf.; 81: Psychotria viridis.tw,kf.; 82: Anadenanthera peregrina.tw,kf.; 83: mescalin*.tw,kf.; 84: peyote.tw,kf.; 85: peyotl.tw,kf.; 86: Lophophora williamsii.tw,kf.; 87: trimethoxyphenethylamine.tw,kf.; 88: Ketamine.tw,kf.; 89: Esketamine.tw,kf.; 90: spravato.tw,kf.; 91: katamine.tw,kf.; 92: ketolar.tw,kf.; 93: S-ketamine.tw,kf.; 94: R-ketamine.tw,kf.; 95: Arketamine.tw,kf.; 96: MDMA.tw,kf.; 97: n methyl 3,4 methylenedioxyamphetamine.tw,kf.; 98: ecstasy.tw,kf.; 99: methylenedioxymethamphetamine.tw,kf.; 100: 3,4-Methylenedioxymethamphetamine.tw,kf.; 101: MDMA-assisted psychotherapy.tw,kf.; 102: 3,4 methylenedioxyamphetamine.tw,kf.; 103: methylene dioxyamphetamine.tw,kf.; 104: methylenedioxyamphetamine.tw,kf.; 105: Ibogaine.tw,kf.; 106: Iboga.tw,kf.; 107: endabuse.tw,kf.; 108: 5-MeO-DMT.tw,kf.; 109: Methoxydimethyltryptamine*.tw,kf.; 110: Entactogen*.tw,kf.; 111: 5 methoxy n,n dimethyltryptamine.tw,kf.; 112: 5 methoxydimethyltryptamine.tw,kf.; 113: 5 methoxy dmt.tw,kf.; 114: Salvia divinorum.tw,kf.; 115: Diviners sage.tw,kf.; 116: Phencyclidine.tw,kf.; 117: Phencyclidin.tw,kf.; 118: phenycyclidine.tw,kf.; 119: phenylcyclidine.tw,kf.; 120: Angel dust.tw,kf.; 121: sernyl.tw,kf.; 122: Bufotenin.tw,kf.; 123: Bufotenine.tw,kf.; 124: dimethylserotonin.tw,kf.; 125: 2C-B.tw,kf.; 126: 2C-I.tw,kf.; 127: 2C-E.tw,kf.; 128: 2C-T-7.tw,kf.; 129: 2C-T-2.tw,kf.; 130: 2C-C.tw,kf.; 131: 2C-P.tw,kf.; 132: 2C-T-4.tw,kf.; 133: Argyreia nervosa.tw,kf.; 134: Diisopropyltryptamine.tw,kf.; 135: 5 methoxy n,n diisopropyltryptamine.tw,kf.; 136: foxy methoxy.tw,kf.; 137: 4 iodo 2,5 dimethoxyphenylisopropylamine.tw,kf.; 138: Methylone.tw,kf.; 139: 2,5-Dimethoxy-4-Methylamphetamine.tw,kf.; 140: 25I-NBOMe.tw,kf.; 141: 5-MeO-aMT.tw,kf.; 142: 4-AcO-DMT.tw,kf.; 143: 5-MeO-MiPT.tw,kf.; 144: Bromo-DragonFLY.tw,kf.; 145: AL-LAD.tw,kf.; 146: Central Nervous System Stimulant*.tw,kf.; 147: cns stimulant*.tw,kf.; 148: Central stimulant*.tw,kf.; 149: Stimulant-use disorder*.tw,kf.; 150: Stimulant-related disorder*.tw,kf.; 151: Psychostimulant.tw,kf.; 152: Cocaine.tw,kf.; 153: Crack cocaine.tw,kf.; 154: cocain.tw,kf.; 155: Cocaine abuse.tw,kf.; 156: Cocaine addiction.tw,kf.; 157: Cocaine dependen*.tw,kf.; 158: Cocaine related disorder*.tw,kf.; 159: Cocaine-use disorder.tw,kf.; 160: Amphetamines.tw,kf.; 161: Amphetamine-related disorder*.tw,kf.; 162: Amphetamine abuse.tw,kf.; 163: Amphetamine addiction.tw,kf.; 164: Amphetamine dependen*.tw,kf.; 165: Amphetamine-use disorder*.tw,kf.; 166: Amphetamine.tw,kf.; 167: Amfetamine.tw,kf.; 168: Adderall.tw,kf.; 169: levoamphetamine.tw,kf.; 170: adzenys.tw,kf.; 171: Amphetamin.tw,kf.; 172: amphethamine.tw,kf.; 173: Benzedrine.tw,kf.; 174: centramine.tw,kf.; 175: evekeo.tw,kf.; 176: levamfetamine.tw,kf.; 177: levamphetamine.tw,kf.; 178: levoamphetamine.tw,kf.; 179: Methamphetamine.tw,kf.; 180: crystal meth.tw,kf.; 181: deoxyephedrine.tw,kf.; 182: Desoxyephedrine.tw,kf.; 183: desoxyn.tw,kf.; 184: metamfetamine.tw,kf.; 185: methylamphetamine.tw,kf.; 186: methedrine.tw,kf.; 187: pervitin.tw,kf.; 188: Methamphetamine dependenc*.tw,kf.; 189: methamphetamine addiction.tw,kf.; 190: Dextroamphetamine.tw,kf.; 191: Dexamfetamine.tw,kf.; 192: Dexedrine.tw,kf.; 193: dextro amphetamine sulfate.tw,kf.; 194: dextrostat.tw,kf.; 195: Lisdexamfetamine Dimesylate.tw,kf.; 196: elvanse.tw,kf.; 197: lisdexamfetamine.tw,kf.; 198: Vyvanse.tw,kf.; 199: elvanse.tw,kf.; 200: venvanse.tw,kf.; 201: parachloroamphetamine.tw,kf.; 202: chloramphetamine.tw,kf.; 203: chloroamphetamine.tw,kf.; 204: 4-Fluoroamphetamine.tw,kf.; 205: Nicotine.tw,kf.; 206: Nicotine bitartrate.tw,kf.; 207: nicotine tartrate.tw,kf.; 208: Tobacco.tw,kf.; 209: habitrol.tw,kf.; 210: nicabate.tw,kf.; 211: nicoderm.tw,kf.; 212: nicopass.tw,kf.; 213: nicopatch.tw,kf.; 214: Nicorette.tw,kf.; 215: nicotin.tw,kf.; 216: nicotinell.tw,kf.; 217: nicotrol.tw,kf.; 218: niquitin.tw,kf.; 219: Nicotine-use disorder.tw,kf.; 220: Tobacco-use disorder.tw,kf.; 221: Nicotine addiction*.tw,kf.; 222: Tobacco addiction*.tw,kf.; 223: Nicotine dependen*.tw,kf.; 224: Tobacco dependen*.tw,kf.; 225: Nicotine abuse.tw,kf.; 226: Tobacco abuse.tw,kf.; 227: Caffeine.tw,kf.; 228: Coffee.tw,kf.; 229: Yerba mate.tw,kf.; 230: Ilex paraguariensis.tw,kf.; 231: vivarin.tw,kf.; 232: caffein.tw,kf.; 233: percoffedrinol.tw,kf.; 234: coffeine.tw,kf.; 235: Methylphenidate.tw,kf.; 236: centedrin.tw,kf.; 237: concerta.tw,kf.; 238: daytrana.tw,kf.; 239: equasym.tw,kf.; 240: metadate.tw,kf.; 241: methylin.tw,kf.; 242: phenidylate.tw,kf.; 243: Ritalin.tw,kf.; 244: ritalina.tw,kf.; 245: ritaline.tw,kf.; 246: rubifen.tw,kf.; 247: adhansia.tw,kf.; 248: aptensio.tw,kf.; 249: biphentin.tw,kf.; 250: cotempla.tw,kf.; 251: foquest.tw,kf.; 252: jornay.tw,kf.; 253: medicebran.tw,kf.; 254: medikinet.tw,kf.; 255: methylfenidate.tw,kf.; 256: methylphenidylacetate.tw,kf.; 257: methylphenydate.tw,kf.; 258: "prc 063".tw,kf.; 259: quasym.tw,kf.; 260: quillichew.tw,kf.; 261: quillivant.tw,kf.; 262: Dexmethylphenidate.tw,kf.; 263: dexmethylphenidate hydrochloride.tw,kf.; 264: focalin.tw,kf.; 265: ritadex.tw,kf.; 266: mazanor.tw,kf.; 267: mazindol.tw,kf.; 268: sanorex.tw,kf.; 269: phenoxazole.tw,kf.; 270: tradon.tw,kf.; 271: phenmetrazine.tw,kf.; 272: preludin.tw,kf.; 273: dexphenmetrazine.tw,kf.; 274: Cathinone.tw,kf.; 275: Khat.tw,kf.; 276: Synthetic Cathinone*.tw,kf.; 277: Psychoactive bath salts.tw,kf.; 278: Methylenedioxypyrovalerone.tw,kf.; 279: MDPV.tw,kf.; 280: Katinon.tw,kf.; 281: Catinon.tw,kf.; 282: Propylhexedrine.tw,kf.; 283: Benzedrex.tw,kf.; 284: Obesin.tw,kf.; 285: Mephedrone.tw,kf.; 286: Ethylphenidate.tw,kf.; 287: cannabi*.tw,kf.; 288: cannabis.tw,kf.; 289: bhang.tw,kf.; 290: hashish*.tw,kf.; 291: marihuana*.tw,kf.; 292: marijuana*.tw,kf.; 293: medical marihuana*.tw,kf.; 294: Cannabis-use disorder.tw,kf.; 295: Marijuana-use disorder.tw,kf.; 296: Cannabis abuse*.tw,kf.; 297: Marijuana abuse*.tw,kf.; 298: Cannabis dependenc*.tw,kf.; 299: Marijuana dependenc*.tw,kf.; 300: Cannabis addiction.tw,kf.; 301: Marijuana addiction.tw,kf.; 302: Anandamide.tw,kf.; 303: Cannabinol.tw,kf.; 304: Delta8-tetrahydrocannabinol.tw,kf.; 305: Delta9-tetrahydrocannabinol.tw,kf.; 306: Dronabinol.tw,kf.; 307: Levonantradol.tw,kf.; 308: Methanandamide.tw,kf.; 309: Nabiximol*.tw,kf.; 310: Nantradol.tw,kf.; 311: Tetrahydrocannabinol.tw,kf.; 312: Tetrahydrocannabinol derivative*.tw,kf.; 313: THC.tw,kf.; 314: Delta-9-THC.tw,kf.; 315: Delta-9-tetrahydrocannabinol.tw,kf.; 316: Delta-1-THC.tw,kf.; 317: Delta-1-tetrahydrocannabinol.tw,kf.; 318: 1 or 2 or 3 or 4 or 5 or 6 or 7 or 8 or 9 or 10 or 11 or 12 or 13 or 14 or 15 or 16 or 17 or 18 or 19 or 20 or 21 or 22 or 23 or 24 or 25 or 26 or 27 or 28 or 29 or 30 or 31 or 32 or 33 or 34 or 35 or 36 or 37 or 38 or 39 or 40 or 41 or 42 or 43 or 44 or 45 or 46 or 47 or 48 or 49 or 50 or 51 or 52 or 53 or 54 or 55 or 56 or 57 or 58 or 59 or 60 or 61 or 62 or 63 or 64 or 65 or 66 or 67 or 68 or 69 or 70 or 71 or 72 or 73 or 74 or 75 or 76 or 77 or 78 or 79 or 80 or 81 or 82 or 83 or 84 or 85 or 86 or 87 or 88 or 89 or 90 or 91 or 92 or 93 or 94 or 95 or 96 or 97 or 98 or 99 or 100 or 101 or 102 or 103 or 104 or 105 or 106 or 107 or 108 or 109 or 110 or 111 or 112 or 113 or 114 or 115 or 116 or 117 or 118 or 119 or 120 or 121 or 122 or 123 or 124 or 125 or 126 or 127 or 128 or 129 or 130 or 131 or 132 or 133 or 134 or 135 or 136 or 137 or 138 or 139 or 140 or 141 or 142 or 143 or 144 or 145 or 146 or 147 or 148 or 149 or 150 or 151 or 152 or 153 or 154 or 155 or 156 or 157 or 158 or 159 or 160 or 161 or 162 or 163 or 164 or 165 or 166 or 167 or 168 or 169 or 170 or 171 or 172 or 173 or 174 or 175 or 176 or 177 or 178 or 179 or 180 or 181 or 182 or 183 or 184 or 185 or 186 or 187 or 188 or 189 or 190 or 191 or 192 or 193 or 194 or 195 or 196 or 197 or 198 or 199 or 200 or 201 or 202 or 203 or 204 or 205 or 206 or 207 or 208 or 209 or 210 or 211 or 212 or 213 or 214 or 215 or 216 or 217 or 218 or 219 or 220 or 221 or 222 or 223 or 224 or 225 or 226 or 227 or 228 or 229 or 230 or 231 or 232 or 233 or 234 or 235 or 236 or 237 or 238 or 239 or 240 or 241 or 242 or 243 or 244 or 245 or 246 or 247 or 248 or 249 or 250 or 251 or 252 or 253 or 254 or 255 or 256 or 257 or 258 or 259 or 260 or 261 or 262 or 263 or 264 or 265 or 266 or 267 or 268 or 269 or 270 or 271 or 272 or 273 or 274 or 275 or 276 or 277 or 278 or 279 or 280 or 281 or 282 or 283 or 284 or 285 or 286 or 287 or 288 or 289 or 290 or 291 or 292 or 293 or 294 or 295 or 296 or 297 or 298 or 299 or 300 or 301 or 302 or 303 or 304 or 305 or 306 or 307 or 308 or 309 or 310 or 311 or 312 or 313 or 314 or 315 or 316 or 317; 319: natural language processing/; 320: language processing/; 321: sentiment analysis/; 322: Natural Language Process*.tw,kf.; 323: Language Process*.tw,kf.; 324: Sentiment Analys*.tw,kf.; 325: Semantic Analys*.tw,kf.; 326: Linguistic Analys*.tw,kf.; 327: Keyword extract*.tw,kf.; 328: Part-of-speech.tw,kf.; 329: Parts-of-speech.tw,kf.; 330: Parts of speech.tw,kf.; 331: POS tag*.tw,kf.; 332: 319 or 320 or 321 or 322 or 323 or 324 or 325 or 326 or 327 or 328 or 329 or 330 or 331; 333: 318 and 332;

# References

Agurto C, Cecchi GA, Norel R, et al. (2020) Detection of acute 3,4-methylenedioxymethamphetamine (MDMA) effects across protocols using automated natural language processing. *Neuropsychopharmacology* 45(5). Springer Nature: 823–832.

Bedi G, Cecchi GA, Slezak DF, et al. (2014) A window into the intoxicated mind? Speech as an index of psychoactive drug effects. *Neuropsychopharmacology* 39(10). Nature Publishing Group: 2340–2348.

GraphPad Prism (2024) 10.20.0. San Diego: GraphPad.

Sanz C, Zamberlan F, Erowid E, et al. (2018) The experience elicited by hallucinogens presents the highest similarity to dreaming within a large database of psychoactive substance reports. *Frontiers in Neuroscience* 12(JAN). Frontiers Media S.A.

Wardle MC and De Wit H (2014) MDMA alters emotional processing and facilitates positive social interaction. *Psychopharmacology* 231. Springer Verlag: 4219–4229.

Zeidenberg P, Clark WC, Jaffe J, et al. (1973) Effect of Oral Administration of Δ9 Tetrahydrocannabinol on Memory, Speech, and Perception of Thermal Stimulation: Results With Four Normal Human Volunteer Subjects. Preliminary Report. *Comprehensive Psychiatry* 14(6): 549–556.

 supplementary table and Figure Legends

**Supplementary Table 1** Pooled Demographic Characteristics. *A random-effect model was used to pool participants’ mean ages and years of education. For cohorts that were used in multiple publications (e.g. as the training cohort in one study and the validation cohort in another study (Bedi et al., 2014; Wardle and De Wit, 2014), those participants were only counted once. ^I^Number of studies with data fit for pooled analysis;* ^II^*or “Caucasian”;* ^III^*or “African American”;* ^IV^*other and/or mixed race.*

**Supplementary Figure 1** Risk of bias assessments. *From top to bottom: risk of bias for cross-sectional studies, risk of bias for non-randomized trials, and risk of bias for randomized crossover trials included in the review. ^I^For Agurto et al., 2020, we report the risk of bias for the main cohort of 31 healthy adults; ^II^Wardle and De Wit, 2014 and ^III^Bedi et al., 2014 are both independently included in the review, but the data from these studies were also used in Agurto et al., 2020 as “Independent Datasets 1 and 2” for model testing.*
