# Supplementary material for: Quantitative natural language processing markers of psychoactive drug effects: A pre-registered systematic review
Source: J Psychopharmacol. 2025 Feb 16;39(9):940–9. doi: 10.1177/02698811251319455 (PMC12371134; doi:10.1177/02698811251319455)
Supplement: sj-docx-3-jop-10.1177_02698811251319455 – Supplemental material for Quantitative natural language processing markers of psychoactive drug effects: A pre-registered systematic review [file sj-docx-3-jop-10.1177_02698811251319455.docx]

**Supplementary Figure 1** Risk of bias assessments


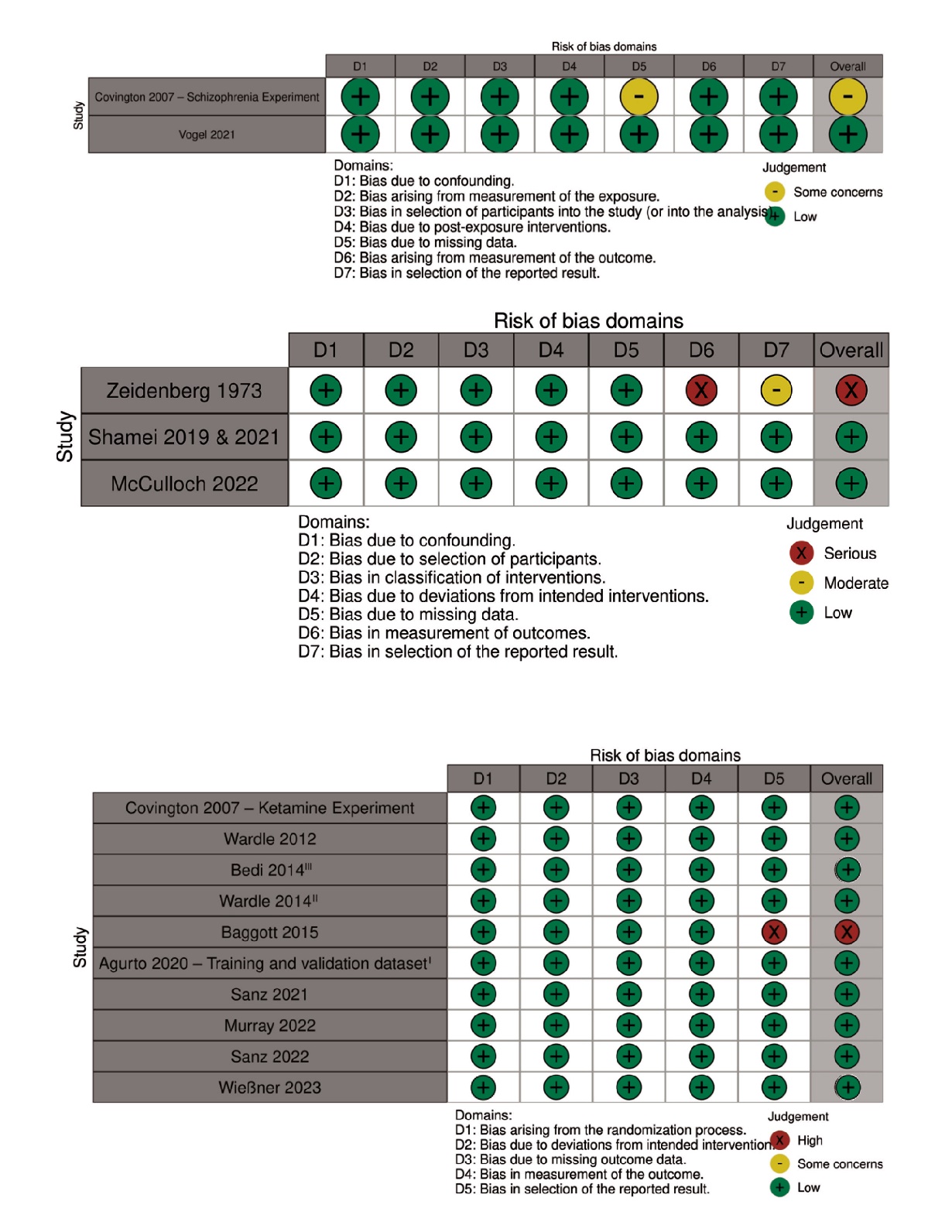


*From top to bottom: risk of bias for cross-sectional studies, risk of bias for non-randomized trials, and risk of bias for randomized crossover trials included in the review. ^I^For Agurto 2020* [5]*, we report the risk of bias for the main cohort of 31 healthy adults; ^II^Wardle 2014* [3] *and ^III^Bedi 2014* [4] *are both independently included in the review, but the data from these studies were also used in Agurto 2020* [5] *as “Independent Datasets 1 and 2” for model testing.*
